# Editorial: Insights in applied genetic epidemiology 2025

**DOI:** 10.3389/fgene.2026.1801003

**Published:** 2026-02-16

**Authors:** Hui-Qi Qu, M. Geoffrey Hayes

**Affiliations:** 1 The Center for Applied Genomics, Children’s Hospital of Philadelphia, Philadelphia, PA, United States; 2 Division of Endocrinology, Metabolism, and Molecular Medicine, Department of Medicine, Northwestern University Feinberg School of Medicine, Chicago, IL, United States; 3 Center for Genetic Medicine, Northwestern University Feinberg School of Medicine, Chicago, IL, United States; 4 Department of Anthropology, Northwestern University, Evanston, IL, United States

**Keywords:** applied genetic epidemiology, cross-ancestry GWAS, genetic pleiotropy, multi-trait analysis, polygenic risk scores (PRS)

Over the past two decades, applied genetic epidemiology has used genome-wide data to study complex diseases. The field has moved from finding single loci to modeling polygenic risk across the genome. Polygenic and cross-trait methods improve discovery and enable systematic cross-trait analyses. They also expose translational constraints: limited replication, poor cross-ancestry portability of polygenic risk scores (PRS), and the need for calibration/validation in the target population. The Insights in Applied Genetic Epidemiology 2025 Research Topic brings together seven papers on cardiovascular disease (CVD), asthma, gastroesophageal reflux disease (GERD), and cancer spanning multi-trait analyses, risk prediction studies, and reviews of variant classes and biological modifiers.

A consistent theme across the Research Topic is the shift from a locus-by-locus narrative toward quantitative modeling approaches that still preserve biological interpretability. The goal is to use genome-wide statistics to support stratification and study design, while using interpretable signals to prioritize pathways and intervention points. The practical question is not whether complex disease risk is polygenic; it is which modeling framework the field adopts, what it omits, and how those omissions motivate study design and validation. The field is shifting from reductionist discovery of single loci to a discipline organized around these frameworks and tested across contexts and populations. Many investigators enter the field with a clinically shaped mechanistic instinct: identify a key mechanism and act on it. Others enter the field from a population genetics background which bringing a second view in which risk is distributed, causality is layered, and useful models often summarize complexity while still pointing toward biology. Neither perspective invalidates the other, and both make invaluable contributions to the development of the field.

Several contributions treat correlation and comorbidity as modellable features rather than nuisances to adjust away. In CVD, Zhong et al. integrate large-scale East Asian GWAS summary statistics across myocardial infarction, heart failure, atrial fibrillation, arrhythmia, and cardiometabolic traits. They combine genome-wide and local genetic correlation with multi-trait association testing and functional annotation, and report tissue-relevant enrichment patterns in cardiovascular contexts. Chen et al. analyze colorectal cancer in BioBank Japan using multi-trait analysis, along with genome-wide and local genetic correlation and heritability enrichment across chromatin states and tissue types. Their results illustrate how joint modeling can improve locus discovery and can provide convergent evidence through colocalization and regulatory annotation. Extending cross-trait thinking to a clinically familiar interface, Gao et al. investigate genetic enrichment between GERD and psychiatric disorders in East Asian populations using linkage disequilibrium (LD) score regression and conditional false discovery rate (FDR). Their approach illustrates how pleiotropy-aware methods can increase locus discovery and motivate biological hypotheses relevant to gut–brain signaling pathways. The shared message is methodological and practical: multi-trait analysis operationalizes overlap among phenotypes and improves discovery and interpretation, particularly when ancestry-specific datasets remain limited in scale for some traits.

This perspective is particularly clear in the way PRS appear in the Research Topic. PRS is often presented as a prediction tool, yet its deeper utility is frequently analytic. It helps define who carries higher genetic liability, exposes heterogeneity hidden within clinical labels, and shows what remains unexplained once common-variant additive signal saturates. The most informative outputs are not always those with the highest Areas under the Curve (AUCs), which should garner the attention of investigators moving forward regardless of their phenotype of interest. Mismatches can be more revealing, especially when performance changes across ancestry, subtype, age, or sex. The residual risk can point to missing biology, measurement error, or non-portable effect estimates.

Risk prediction is addressed with a similarly applied, pragmatic stance. Gao et al. evaluate three PRS approaches for lung cancer in a small Chinese cohort, comparing a previously proposed 19-SNP score with genome-wide Bayesian approaches (PRS-CS) and cross-ancestry integration (PRS-CSx). Their results emphasize the importance of ancestry-matched inputs and show where performance remains limited by sample size and subtype heterogeneity. Velasco Parra et al. report clinical validation of an integrated risk assessment approach for sporadic breast cancer in Colombia. They show that PRS is most informative when combined with established predictors such as family history and imaging-derived breast density, improving discrimination beyond any single component. These two studies highlight a translational pattern: PRS gains clinical meaning when embedded in a multivariable framework, and its performance must be assessed with attention to calibration and the population in which it will be used. They also underscore a broader lesson: plateaus and mismatches can be informative, pointing to missing model classes such as rare variation, non-additive effects, phenotype heterogeneity or misclassification, and context-dependent modifiers.

The Research Topic reinforces that “genome-wide” should not remain synonymous with common SNPs. Colombage et al. review structural variants (SVs) in CVD and show how whole-genome sequencing enables comprehensive detection. They summarize SV classes and whole genome sequence (WGS)-based SV calling approaches and tools, emphasizing method-dependent accuracy and the need for benchmarking and harmonized pipelines. The review also notes that SV-focused CVD studies remain limited, and it argues that integrating SV data with population-scale epidemiology and risk prediction is a clear next step for translation.

Biological context appears most directly in the review by Peng et al. on sex differences in asthma. It summarizes omics evidence and emphasizes the gap between emerging biological understanding and guideline-level implementation. Conceptually, modeling sex only as an adjustment covariate can implicitly restrict sex differences to a main-effect shift within the fitted model. Evidence across diseases suggests that effect heterogeneity by sex can be biologically meaningful and can influence both inference and prediction. If context modifies pathways and clinical trajectories, it can also modify how genetic signals manifest and how risk models behave. Turning this into practice will require study designs and reporting standards that can detect heterogeneous effects, evaluate calibration in relevant strata, and connect biological signals to actionable clinical questions.

The seven papers in Insights in Applied Genetic Epidemiology 2025 do not attempt to define the field, yet they capture a direction for the field. Applied genetic epidemiology is increasingly organized around genome-wide polygenic modeling, multi-trait inference, and integrated risk prediction, with growing attention to portability across populations and to effect modifiers such as variant class and sex. Progress will come from combining these elements carefully: multi-trait inference coupled to functional interpretation, genetic risk modeled alongside clinical and environmental information, and study designs built to test portability and heterogeneity rather than ignore them.

